# Bethlem Hospital—Dr. Wright

**Published:** 1831-04-01

**Authors:** 


### VIII.

### BETHLEM HOSPITAL—DR. WRIGHT.

We are sorry to perceive that a few indiscretions and unguarded levities, on the part of the talented apothecary of the above institution, Dr. Wright, have been magnified by a pack of ignorant and malicious menials, and by, we cannot help fancying, a most partial tribunal of judges, into a long train of enormous charges and delinquencies, ending in the expulsion of Dr. Wright from his situation in Bethlem. The gravamen of the accusation against the unfortunate apothecary, appears to be the fact, or rather the suspicion, resting on the testimonies of some discarded and disappointed servants, that Dr. Wright had been "the worse for liquor," some eight years ago, and "once since that time," some eighteen months ago! There are some episodes indeed, to the grand drama, which swell the tide of fortune against the Doctor. Thus he is supposed to have been the principal, or a powerful auxiliary to the decapitation of Margaret Nicholson—after her death—and to have winked at the artifice of an accomplice in the crime, who made an artificial head filled with saw-dust, and afterwards turned informer against the phrenological enquirers. These deady sins would probably have been pardoned, had not the Doctor taken some liberties with one or two of those vestals who guard the weak intellects of the Bedlamites. One of these vestals swore, or asserted, that she was not married, but, on being cross-questioned, deposed to the contrary—a trifling inconsistency, which made no impression on the impartial judges, who listened with eagerness to every thing against the accused, but passed over unnoticed all that militated in his favour. The whole investigation, indeed, is a series of "leading questions" on the part of the committee, by which every thing that they apparently wished to come forth, was put, cut and dry, into the mouths of the witnesses! They must have been stupid dolts who could not have seen that the object of their being cited before this dread tribunal, was, not to tell the truth, the whole truth, and nothing but the truth—but, on the contrary, to furnish matter of accusasation against Dr. Wright, all other testimony being evidently deemed irrelevant, and unnecessary.

We have admitted, in the outset, that Dr. Wright has probably been unguarded; but the charges have been magnified and distorted in such a manner that we cannot but conclude that he has been very harshly dealt with. The mischief, however, is done, and Dr. Wright, with a family of children, is turned adrift, and will probably be ruined by this decision of the committee of investigation. We are sorry for it!

---

